# MRI and the Critical Care Patient: Clinical, Operational, and Financial Challenges

**DOI:** 10.1155/2023/2772181

**Published:** 2023-06-06

**Authors:** Barbara McLean, Douglas Thompson

**Affiliations:** ^1^Division of Emergency Services and Critical Care, Grady Health System, Atlanta, GA, USA; ^2^Open Box, LLC, Mesa, AZ, USA

## Abstract

Neuroimaging in conjunction with a neurologic examination has become a valuable resource for today's intensive care unit (ICU) physicians. Imaging provides critical information during the assessment and ongoing neuromonitoring of patients for toxic-metabolic or structural injury of the brain. A patient's condition can change rapidly, and interventions may require imaging. When making this determination, the benefit must be weighed against possible risks associated with intrahospital transport. The patient's condition is assessed to decide if they are stable enough to leave the ICU for an extended period. Intrahospital transport risks include adverse events related to the physical nature of the transport, the change in the environment, or relocating equipment used to monitor the patient. Adverse events can be categorized as minor (e.g., clinical decompensation) or major (e.g., requiring immediate intervention) and may occur in preparation or during transport. Regardless of the type of event experienced, any intervention during transport impacts the patient and may lead to delayed treatment and disruption of critical care. This review summarizes the commentary on the current literature on the associated risks and provides insight into the costs as well as provider experiences. Approximately, one-third of patients who are transported from the ICU to an imaging suite may experience an adverse event. This creates an additional risk for extending a patient's stay in the ICU. The delay in obtaining imaging can negatively impact the patient's treatment plan and affect long-term outcomes as increased disability or mortality. Disruption of ICU therapy can decrease respiratory function after the patient returns from transport. Because of the complex care team needed for patient transport, the staff time alone can cost $200 or more. New technologies and advancements are needed to reduce patient risk and improve safety.

## 1. Introduction

Standard of care (SOC) for patients in the intensive care unit (ICU) requires proper diagnosis, baseline measurements, and establishing progression of care so that the patient spends the shortest time necessary in the ICU [1, 2]. Imaging in conjunction with neurological examination is often necessary to help the care team diagnose, monitor, and treat the patient. For neurocritical care patients, computed tomography (CT) and magnetic resonance imaging (MRI) are the most commonly used imaging modalities, with CT being used more often because accessibility can be faster and cheaper [3, 4]. High-field MRI can carry certain inherent challenges related to susceptibility to ferromagnetic materials or contraindications due to the equipment needed by an ICU patient during MRI [5]. Outside of any practical limitations related to MRI, this imaging method is preferred in the neurocritical care setting for diagnosis, intervention, and ongoing management of patients because of its enhanced sensitivity and soft tissue resolution.

**Figure 1:**
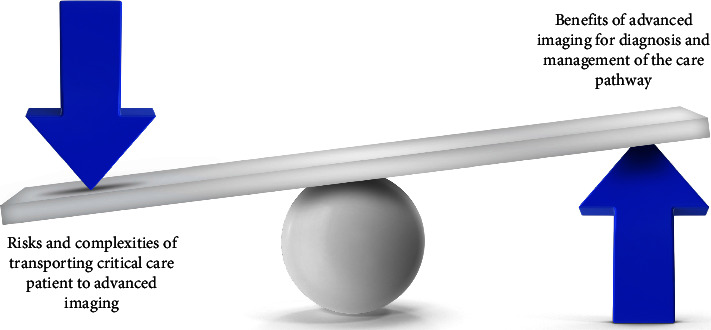
Benefits and risks and complexities of advanced imaging for critical care patients.

**Figure 2:**
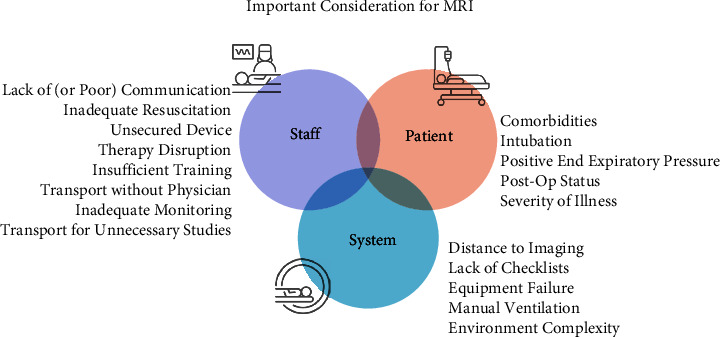
Important considerations for transporting patient to and from the MRI suite.

Intrahospital transport of the patient to the MRI suite from the ICU and back is time-consuming. It can become costly and dangerous; therefore, the risks to the patient must be weighed carefully by the transport team and treating physician (Figures 1 and 2) [1, 2, 6–10].

From a patient safety and quality perspective, transport for neuroimaging while the patient is critically ill requires consideration of several issues. First, what is the potential damage or risk to the patient during intrahospital transport? Second, what is the risk to the patient being away from the prescribed SOC in the ICU? Third, how will delays in obtaining imaging impact needed care and extend ICU and hospital lengths of stay? From the physician perspective, the physical findings of a physical exam combined with MRI may serve as a decision point for the best treatment option. Thus, precious time delays in obtaining neuroimaging results are compounded by the wait for a transport team to prepare the patient, obtain a scan, and return the patient to the ICU setting, all of which can take hours. Fourth, what are the potential costs related to neuroimaging and intrahospital transport? Fifth, what are the costs related to the hospital system for adverse events (AEs) during intrahospital transport and how does this disruption impact patient care?

A significant challenge while the patient is being imaged in conventional high-field MRI is the patient is physically separated from the nurse who is supposed to monitor the patient during the scan. This results in limited visibility of the patient during the scan, and the nurse may not be able to quickly access the patient if an emergency arises. The potential for life-threatening emergencies increases because the staff is unable to hear any alarms on pumps, ventilators, or other life support equipment. Additionally, there can be a delay in accessing the patient during an emergency because only a handful of people previously cleared can access the patient in MRI. If the patient requires additional support personnel, they will need to be moved to the safest zone to minimize the risk of having to shut down the magnet. Emergency interventions to a patient during a scan can only be performed by personnel who know the direct route to the patient.

The evidence that has been collected regarding AEs during transport and in the MRI tends to differ from what is reported at the hospital level because policy requires AEs to be self-reported by staff, which may not occur. The problems with the current SOC and staffing shortages, the evidence of AEs recorded during transport and in the MRI, the risk factors for AEs during transport, risk associated with delay of imaging and delay of care, and the financial implications of these issues are the focus of discussion. Solutions to these problems are not the focus of discussion but are suggested when appropriate and additional research is needed to provide steppingstones for improvement.

## 2. The Problem Today

**Figure 3:**
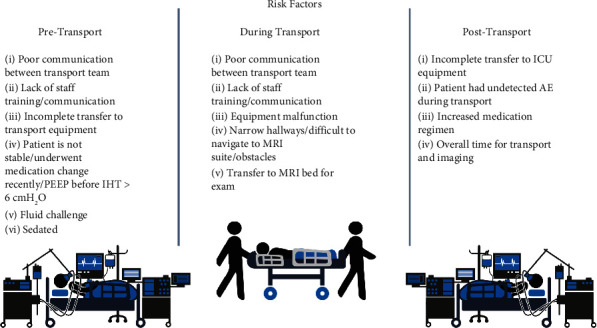
Risk factors during patient transport for adverse events and outcomes.

Globally and more specifically in the United Kingdom and US-based ICUs, the standard patient to provider ratio is two to one; although for specific situations where patient acuity is worse, the ratio can be increased to one to one [11–16]. However, research suggests that it is still difficult to quantify the change in patient care when the ratio is increased as there is not always improvement in patient outcomes [11, 13, 16]. Thus, when a provider orders imaging which requires transporting patients outside of the ICU, if the hospital does not have a dedicated transport team, the nurse must find backup coverage to care for their other patients (i.e., when the ratio is two to one). This staffing model creates risk for the other patients in the ICU. For patients who require transport teams, additional staff members are brought in, including a respiratory therapist (RT), a physician (usually a resident), and care technicians, to help prepare the patient for transport (e.g., medications, ventilator, cardiac monitor, and checking vitals) and further navigate through the hospital while monitoring the patient (Figure 3). In a high acuity, high MRI demand center, where patients are even more at risk, the time to prepare for transport, transport to and from the MRI suite, and to get the patient reconnected to equipment in the ICU room ranges from 1 to 2 hours [9, 10]. Accounting for the time from when a physician places an imaging order until the results are obtained can take up 8 hours (Table 1) according to interviews conducted by the author with ICU and radiology staff. There is a wide variation in these cycle times from hospital to hospital and institution to institution, where one study found the average turnaround time from placement of the imaging request to initiation of an MRI scan to be 751 minutes (∼12.5 hours) [17] and with some institutions reporting times from MRI order to result of more than 24 hours. For example, a study by Tiwari et al. [18] suggests that the average waiting time for an inpatient with suspected occult femoral neck fracture for an MRI scan is 23 hours. The wait time for the study or procedure has also been identified as a factor that increases the risk of mishaps during transport [19]. Depending on the exam that is ordered and wait times or delays once arriving at the MRI suite [17, 18, 20], there may be a longer time required for the transport team to be away from their normally assigned positions complicated by the time that the patient is away from therapy and the standard ICU environment. A consideration for ICU managers is whether to backfill for the staff that are part of the transport team. This is largely based on the preference and budget of the hospital, but in the current environment, the severe nursing shortage throughout hospital systems can lead to postponing imaging for patients because there are not enough staff available for transport [21]. The staffing deficit is a problem faced by the US healthcare system broadly as well as individual hospitals and institutions; indeed, solutions to this problem are outside the scope of this discussion.

In the neurocritical care setting, the ordering physician needs to consider the risk to the patient by choosing a CT scan over MRI. Often the risks to the patient from the CT scan itself are not considered; for example, exposure to radiation from CT scans has been found to increase risk for cancer in adult and pediatric patients but lower risk in 60 year olds [22, 23]. These risks may not be at the top of mind for the ICU physician who is trying to answer a clinical question related to the care of their patient.

### 2.1. Risks for ICU Patients Being Transported to MRI

There are numerous risks for ICU patients being transported to the MRI suite that span the patient, staff, and hospital system (Figures 2 and 3) [8]. The transport process is complex and requires team work as well as defined roles and consistent communication to ensure the safety and health of the patient. Multiple studies have found that, even with protocols and defined roles, staff communication during transport is critical to patient safety [24–27], but communication is challenging and inconsistent in many hospitals, making the transport process more dangerous for the patient [6–9, 28, 29]. As previously discussed, there are increased risks for the patients that remain in the ICU or other units when staff on the transport team are pulled, often leaving insufficient staff to backfill.

During transport, numerous studies have reported AEs ranging from 22% to 79% of cases (Table 2) [6, 7, 10, 19, 30, 31]. We use 33% as the base rate for this estimate, meaning that one-third of all patients have an AE during transport. According to Waydhas [1] and Ott et al. [32], these AEs result from an interruption in monitoring, brief disconnection of intravenous medications required for maintenance of hemodynamic stability, and poor ventilation, with life-threatening events occurring in up to 6–8% of transportations. These AEs can be patient- or equipment-related and classified as minor or major. Of note, the MRI environment is stressful for patients sensitive to loud noises and confined spaces or who may be experiencing hypoactive delirium [33, 34], which may also affect clinical workflow and may increase the need to repeat a sequence, causing more delays [35].

Several studies have conducted univariate and multivariate analyses to determine the most important risk factors in patients who experience an AE during transport. Parmentier-Decrucq et al. [6] found in a multivariate analysis that, for patient-related AEs, only positive end-expiratory pressure (PEEP) > 6 cm H_2_O and treatment modification for transport were significant risk factors. Jia et al. [10] found that patient characteristics did not significantly affect the occurrence of critical patient-related AEs during transport. However, clinical characteristics before transport, including Acute Physiology and Chronic Health Evaluation (APACHE II) score ≥20, partial pressure of oxygen in arterial blood (PaO_2_) <80, abnormal lactate and glucose levels, heart rate <50 or >110, respiratory rate <12 or >25, pulse oximetry <95, and sedation were significant for multivariate analysis [10]. This suggests that use of identification criteria for risk stratification and balancing the risk/benefit ratio before starting transport is important.

The MRI suite itself represents challenges to care related to requirements for modification of ventilation parameters, complex hemodynamic variations, and positional needs for patients with elevated intracranial pressures, as well as limitation of alarm response [36]. There are incidental issues, such as a drop in blood pressure or mucous plug that occur while the patient is in the MRI environment and the provider is outside [36]. With MRI, there is an acute separation from the provider and delay until the exam sequence can be stopped to allow access to the patient to be safely moved to zone 1, which is a challenge for both the patient and provider [36]. This is unique to MRI due to the magnetic field that can be dangerous, unlike other types of imaging where the patient can be rapidly accessed. Patients undergoing MRI have higher rates of needing a rapid response team to assist with medical emergencies that occur during the exam [32, 37]. Additionally, aborted transport of ICU patients to MRI occurred more frequently than for other advanced imaging modalities, suggesting that these patients require more care and evaluation before proceeding with transport [38]. Anesthesiologists, in addition to the provider, face challenges with monitoring the patient while in the MRI environment as there are often equipment disturbances from the magnet, which can require adjustments to ensure that the patient is safe [36]. Critical care patients may require extended stays in the ICU and may require more MRI scans as well as transport [39]. Several studies [2, 8, 27, 31, 40] suggest that it is ideal to minimize the length of transport, to have the imaging suite as close as possible to the ICU or emergency department, and to consider the best route for optimal patient safety. Another case study suggested that intrahospital transport teams plan for determining where to handle events like resuscitation should this happen during transport, for example, is the hallway acceptable or is a clinical area needed [41]. However, minimizing the length of transport and identifying locations midtransport may not always be possible. Rural or smaller hospitals may lack patient monitoring protocols, and hospitals in low-income countries may not have enough staff or technology in place to monitor for AEs during transport, thereby leading to underreporting of AEs [31]. One potential solution to consider is the use of a portable MRI machine that can be used at the patient's bedside. Another option for improving patient safety is to have specially trained transport teams that use a checklist to ensure that risks that can be addressed ahead of transport have been considered and that communication with the imaging suite has been established to minimize wait times and delays [27]. Additional research on the main risk factors for patient safety needs to be conducted.

### 2.2. Risks of Delay for Imaging

For patients with persistent neurological deficit not explained by CT, MRI may provide the information that is critical to determining the next steps. Several studies show that the information obtained via MRI helps improve traumatic brain injury (TBI) outcomes (e.g., mortality) by informing more intensive treatment [42–44]. It follows that delays in obtaining an MRI may have a negative effect on life-saving information for TBI patient outcomes. As described earlier, hospitals have a wide range of turnaround times for ICU MRI results. Even in the best-case scenario, the logistical and clinical challenges of finding an available high-field MRI schedule slot and moving patients from the ICU to the MRI suite and back add at least several hours, and in some cases, more than 24 hours is needed before MRI results become available [17]. Diagnostic delays due to staff shortages, radiology process issues, equipment errors, patient-related rescheduling, and equipment availability are cited as a growing cause of medical negligence claims [45]. A recent study showed that obtaining MRI early is both safe and effective, but its benefits should be weighed against the significant risks of intrahospital transportation to the MRI suite and the high-field MRI exam itself [44]. MRI alternatives, such as a portable MRI machine that can be used to perform a scan at the bedside in the ICU, which avoid intrahospital transportation and high-field scanner scheduling delays, may improve patient outcomes by speeding up MRI results.

### 2.3. Risks of Therapy Interruptions While Undergoing Imaging

In addition to the risks associated with the actual transport of the ICU patient, studies have shown that the patient's condition will worsen because of their removal from the ICU environment and the continuity of care (i.e., deterioration of respiratory function after return from transport) [40, 46, 47]. The critical intensive care patient requires continuous monitoring and precise intravenous medication adjustments. Both of these are impossible to achieve when the patient is in the MRI, compromising safety and impacting recovery [48].

A study by Meng et al. [49] found that there were increased odds of developing a fever (32.94%), tachypnea (79.5%), and hypertension (134%) as a result of medication administration delay averaging 60.8 minutes during ICU nurse shift changes. This suggests that delays in medication administration can have substantial impact and can be extended to patients who are undergoing transport for imaging. Papson et al. [40] noted that infusion interruptions and missed drug administration were unexpected AEs that occurred during transport.

## 3. Potential Costs for ICU Patient Transport

Costs associated with ICU patient transport must be evaluated on several levels. First, there is the cost of the imaging itself, which is absorbed by the hospital under fixed, diagnostic-related grouping-based reimbursement. Second, the staff cost of transport to the MRI suite and back to the ICU is on average $352 based on our calculations, but this does not include the cost and staffing differences in high-risk versus low-risk patients and the extra time and staff that may be needed for higher risk patients for transport (calculated based on nursing, transporter, RT, certified nursing assistant, certified registered nurse anesthetists/anesthesiologists, and physician costs per minute of transport). Conducting MRI on the evening shift or off-hours incurs an additional cost of $100 for these scans.

Prior to the Affordable Care Act, Mello et al. [50] evaluated the cost incurred by hospitals for each AE, where there were 465 injuries that resulted in $1,791,358 in injury-related inpatient care costs (∼$3,852 per injury) that hospitals were unable to recoup [50]. Inflating this figure from 2005 dollars to 2021 dollars (https://www.in2013dollars.com/Medical-care/price-inflation) increases to $6,255 per injury. Payment methods have changed substantially since 2005 and with the approval of the Affordable Care Act, by becoming more fixed and making it harder for hospitals to bill payers for AE costs while also absorbing more of the AE-related costs. This makes the cost figures from this study very conservative for our estimations; therefore, we assume an average cost of $6,255 per AE borne by hospitals. Adler et al. [51] determined that the inpatient cost of harms (e.g., the direct variable cost) for all patients to hospitals is approximately $1,112 ($1,495.53 in 2022 dollars). Based on data from the Kaiser Family Foundation [52] (national average hospital inpatient adjusted expenses per day $2,431.44 (2022)) and Dasta et al. [53] (ICU-ventilated ($12,287.23) and nonventilated ($8,385.63) patient costs per day in 2002 extrapolated to 2022), we estimate the increased costs for ICU patients to be approximately four times that of other hospital patients. This suggests that AEs to ICU patients cost hospitals an average of $5,982.12 per day. The hospital costs for ICU patient AEs were similar using two different approaches, thus supporting the validity of our calculations.

Additional parameters that influence costs associated with ICU patients and the daily cost of an ICU day were considered through evaluation of the additional expense of mechanical ventilation. We used the 2003 cost figures shown by Dasta et al. [53] for the third and subsequent ICU days, with 1/3 of patients mechanically ventilated and 2/3 not ventilated (blended average cost per day of $3,443), and inflated those costs to 2021 for an average cost of $6,083 per day or $253 extra per hour. If more than the typical 1/3 of patients, such as 50–80%, were ventilated, the average cost per hour would be closer to $300. This is supported by other studies that monitored the frequency of transport during an ICU stay [39]. Based on several sources, we estimate that variable costs are only 25% of total costs, so the variable cost per hour of ICU care would be $75. These detailed calculations show that each additional hour a patient is kept in the ICU adds up quickly; therefore, avoiding AEs and other risks during transport to MRI can decrease overall costs for staying in the ICU and treatment. One study in Ireland suggests that time delay in obtaining advanced imaging for patients in the emergency department could reduce the duration of hospitalization, thereby reducing costs [54].

## 4. Patient Experience with Intrahospital Transport

From a patient's perspective, the main objectives for receiving care in the ICU are to reduce treatment delay, length of stay, and morbidity and mortality in the shortest and most efficient manner [55]. Furthermore, the Quadruple Aim by the Institute for Healthcare Improvement, which encompasses care, health, cost, and meaning in work, suggests that improving the patient experience of care and physician experience at hospitals can jointly improve the health of patients as well as reduce the cost of healthcare [56].

## 5. Estimated Burden to the U.S. Healthcare System

Approximately, 5 million patients are admitted to the ICU annually [57], and about 20% of ICU patients were transported for MRI in 2015 [58]. Therefore, with the ICU patient transport AE frequency of approximately 1-in-3 with an estimated average per patient cost to the hospital of $5,982.12, there will be close to 1,000,000 ICU patients annually who will require MRI, and the estimated financial burden to the U.S. healthcare system is $1.79 billion.

## 6. Conclusion

Critical care providers require imaging for their patients to provide a diagnosis or rule out life-threatening complications, yet providers must weigh the risks the patient will be exposed to during the transport for neuroimaging and the time away from the ICU. The risks of not obtaining neuroimaging and transporting the patient can cause delays in making critical care decisions that can lead to complications. The provider must balance the information gained from neuroimaging with the risks of transporting the patient and the time spent outside the ICU. When intrahospital transport is initiated, the cost associated with ICU patient transport to the imaging suite is high and could be further reduced with the introduction of new technology, such as a portable MRI machine or care approaches which limit the need for transport and minimizing AEs during that transport estimated to cost the U.S. healthcare system approximately $1.79 billion annually. Further research is needed to support these advancements.

## Figures and Tables

**Table 1:** Time for transport and MRI scan.

ICU to MRI scan cycle time for conventional MRI (order to result)	Minutes
MRI order to transport initiated	300
Transport initiated to positioned in MRI	65
Positioned in MRI to scan completed	30
Scan completed to patient in bed on unit	75
Patient in bed on unit to result available	30
Total MRI cycle time	500

ICU, intensive care unit; MRI, magnetic resonance imaging.

**Table 2:** Adverse events that can occur during patient transport [8] from the ICU to MRI (adapted from Day).

Category	Adverse event
Airway	(i) Loss
	(ii) Acute obstruction

Breathing	(i) Respiratory arrest
	(ii) Hypoxemia, decreased PaO_2_/FiO_2_ ratio
	(iii) Ventilator-associated pneumonia
	(iv) Tension pneumothorax

Circulation	(i) Cardiac arrest
	(ii) Hemodynamic instability
	(iii) Bleeding
	(iv) Air embolus

Disability (neurological)	(i) Increased intracranial pressure
	(ii) Spinal cord injury destabilization

FiO_2_, fraction of inspired oxygen; ICU, intensive care unit; MRI, magnetic resonance imaging; PaO_2_, partial pressure of oxygen in the arterial blood.

## Data Availability

No data were used to support the findings of this study.
